# Report from the Committee of the House of Commons on Dr. Jenner's Petition, Respecting His Discovery of Vaccine Innoculation

**Published:** 1802-06

**Authors:** 


### *Report from the Committee of the House of Commons on* Dr. JENNER's *Petition, respecting his Discovery of Vaccine Innoculation.*

THE Committee, to whom the Petition of Edward Jenner, Doctor of Physic, was referred, have, pursuant to the Order of the House, examined the Matter thereof; which is divided into Three distinct Heads of Inquiry:

The utility of the discovery itself, which is the foundation of the petition:

The right of the Petitioner to claim the discovery:

The advantage, in point of medical practice, and pecuniary emolument, which he has derived from it.

Upon the first head a number of witnesses of the highest characters, and most extensive experience in the profession, were examined, whose names, with the substance of their respective fpective evidence (ftrongly confirmed by their general practice, as well as by that in their own families) appear in the Appendix; nor was it for want of the teftimony of many other equally refpectable Phyficians and Surgeons, whom the Petitioner was defirous of producing, that many other names are not inferted; but becaufe Your Committee, after having received fo confiderable a body of evidence to the fame purport, and with fo little variation in opinion, thought that his cafe could fuftain no injury in being left to reft upon the concurring depofitions of thofe already examined, who had both the moft ample experience of the facts, and the beft means of forming an opinion upon them. The teftimony alfo of fome perfons not profeffional has been admitted, who could fpeak to occurrences that tend to illuftrate particular points connected with the fubject. The refult, as it appears to Your Committee, which may be collected from the oral teftimony of thefe Gentlemen (with the exception of three of them) is, that the difcovery of Vaccine Inoculation is of the moft general utility, inafmuch as it introduces a milder diforder in the place of the inoculated Small Pox, which is not capable of being communicated by contagion; that it does not excite other humours or diforders in the conftitution; that it has not been known, in any one inftance, to prove fatal; that the Inoculation may be fafely performed at all times of life (which is known not to be the cafe with regard to the inoculation of the Small Pox) in the earlieft infancy, as well as during pregnancy, and in old age; and that it tends to eradicate, and, if its ufe becomes univerfal, muft abfolutely extinguifh one of the moft deftructive diforders by which the human race has been vifited.

The written evidence which is inferted in the Appendix (for Your Committee have judged it proper to make a felection from a great mafs, of what appeared moft important) is more various, but directed to the fame objects: part of it relates to the very extenfive and fuccefsful practice of this mode of inoculation in every quarter of the globe, the efficacy of which does not feem abated by the cold of the northern, nor the heat of the fouthern and tropical climates; and though there are no means of examining the authors from whence fome of thefe atteftations come, it would be an act of injuftice to the Petitioner to exclude thefe important documents, which fhow the confideration in which this difcovery is held, and the benefit with which it has been attended in fo many other countries, to at leaft as great an extent as in our own.

As a comparifon between this new practice, and the inoculated Small Pox, forms a principal confideration in the prefent Inquiry, fome facts with regard to the latter engaged the attention tention of Your Committee, and they have inferted in the Appendix (No. 44) ftatements of the mortality occafioned by the fmall pox in 42 years before Inoculation was practifed in England, and of the 42 years from 1731 to 1772: the refult of which appears to be an increafe of deaths amounting to 17 in every 1000: the general average giving 72 in every 1000 during the firft 42 years, and 89 in the 42 years ending with 1772, fo as to make the whole excefs of deaths in that latter period, 1742. The increafe of mortality is ftated by another witnefs (No. 4) to be as 95 to 70, comparing the concluding 30 years with the firft 30 of the laft century, and the average annual mortality from fmall pox to have been latterly about 2,000; for though individual lives are certainly preferved, and it is true that a fmaller lofs happens in equal numbers who undergo the fmall pox now, than there was formerly; yet it muft be admitted that the general prevalence of inoculation tends to fpread and multiply the difeafe itfelf; of which, though the violence be much abated by the modern mode of treatment, the contagious quality remains in full force. It deferves alfo to be noticed, that the deaths under the inoculated fort of fmall pox, with all the improvements of modern experience, are not inconfiderable; it is ftated by one of the witneffes at about one in every 300 throughout England (No. 35); by another, as about one in every 100 in London (No. 38); while the lofs in the natural fmall pox is probably not lefs than one in fix (No. 37); nor ought it to be overlooked, that miftakes have been known to arife in the inoculated fmall pox, and inftances are cited by fome of the witneffes, in which perfons fuppofed to have gone through the fmall pox by inoculation, have caught it afterwards in the natural way (Nos. 21 and 41); the general laws of vaccine and variolous difeafe are extremely fimilar, and it is not furprifing that they fhould refemble each other in their anomalies.

A fpurious or imperfect fort of Cow Pox having been mentioned in fome of the examinations, Your Committee have been particularly diligent in their inquiries into every individual cafe that came within their notice, where fufpicions had arifen, or facts were alleged, tending to bring into doubt the preventive power of Vaccine Inoculation; and although, for the reafons before given, they have reftricted and abridged the proofs in favour of this practice, they have thought proper to withhold no part of the evidence that has been received relative to the cafes that appear to controvert it; of which it will be obferved that fome (Appendix, Nos. 31, 32, and 33) evidently refolve themfelves into variolous infection, taken previoufly to the vaccine inoculation; others (Appendix, Nos. 33, and 40) into the patient not not having taken the cow pox at all; others again (No. 25) from the vaccine matter being, by want of attention in preferving it, decompofed, or mixed with variolous matter, or from the fluid being taken at too late a period of the puftule; to which laft caufe it feems probable that moft of the errors and dubious cafes are to be referred (No. 20.) All the practitioners agree, that there is no difficulty in diftinguifhing the real diforder from any fpurious or imperfect appearance; and that the regular progrefs of the puftule itfelf, if attended to, cannot be miftaken.

The cafes (Nos. 45, 46, 47, 48, and 49) are not explained by any particular evidence applied to them in a fatisfactory manner: but in leaving them to have fuch weight as they may appear to deferve; Your Committee cannot avoid recurring to the multitude of inftances in which endeavours have been ufed to communicate the fmall pox to patients who have been known to go through the regular vaccine difeafe, in which neither repeated inoculations nor expofure to the moft malignant fmall pox, have been able to produce any effect; Appendix (Nos. 6, 9, 16, 36.)

Upon the fecond head, the whole of the oral depofitions, as well as all the written documents from abroad, are uniform and decifive in favour of Dr. Jenner's claim to originality in the difcovery: but as fome pretenfions have been advanced to a knowledge at leaft of this practice before Dr. Jenner's publications, it may be proper to notice fhortly, what the nature of thofe claims is, and in what manner they bear upon this part of the Petitioner's cafe. The extracts which can be confidered as in any degree material, are contained in Appendix, (Nos. 50, 51, and 52.) The diforder itfelf, and its fpecific property of fecuring againft fmall pox infection, was not a difcovery of Dr. Jenner's, nor of any of thofe whofe writings are referred to: for in various parts of England, in Gloucefterfhire and Devonfhire particularly, there was an opinion of that fort current among the common people employed in Dairies, which the obfervations of inoculators for the fmall pox tended to confirm. It appears not improbable, that in fome very rare inftances this knowledge was carried one ftep farther, and that the cow pox was communicated either by handling the teat, or by inoculation from the animal for the purpofe, and with the intention of fecuring againft the danger of fmall pox: but the practice of which Dr. Jenner afferts himfelf to be the original inventor, is, the inoculation from one human being to another, and the mode of transferring, indefinitely, the vaccine matter without any diminution of its fpecific power, to which it does not appear that any perfon has has ever alleged a title; and the papers and experiments, whatever accuracy of obfervation, and fpirit of refearch, they may evince in their refpective authors, and to whatever extent they may be fuppofed to go, as they were never given to the public, fo neither is there any intimation that they were imparted to Dr. Jenner; nor is it contended that the world became acquainted with this difcovery, by any other means than by the courfe of trials conducted by the Petitioner, and by his ample and unreferved communications.

Upon the laft divifion of the fubject, the evidence of feveral perfons has been received who were acquainted with the medical practice, and former fituation of Dr. Jenner, Appendix (No. 40) which confirm the allegation contained in the Petition, that he has not only reaped no advantage from his difcovery, but that he has been a confiderable lofer by the perfevering attention which he has beftowed upon this one fubject, to the neglect of his other bufinefs, without an opportunity of replacing himfelf in the fituation which a defire of publifhing and diffufing more extenfively, and eftablifhing beyond the reach of controverfy the practice itfelf, induced him to quit. What his gains might probably have been, if he had been folicitous to keep the fecret within his own practice, and that of his own immediate pupils, as far as medical men in great practice themfelves can form a conjectural opinion, may be collected from the teftimonies expreffed in Appendix (Nos. 35 to 43) in which no more than juftice is done to the liberality and public fpirit of the Petitioner, in confidering the propagation and extenfion of this important difcovery, and in rendering it rather of univerfal utility to the human race, than of emolument to himfelf.

Dr. Edward Jenner begged leave to fubmit to the Committee, vouchers from correfpondents in various parts of the globe, referring to at leaft one hundred thoufand cafes. Of thefe teftimonials Your Committee have felected the moft important, and annexed them in the Appendix to this Report.

---

### *Appendix to the Report from the Committee.*

The Evidence delivered by each person is numbered for the sake of reference; but we give them in the order they are referred to in the Report, not in the order of the Appendix.

Dr. BLANE, F. R. S. and one of the Commiffioners of fick and wounded Seamen, firft heard of this difcovery about ten years ago, but could not give credit to what feemed fo extraordinary and romantic, but ftill he did not defift from making inquiries, as fome of his children has fuffered much from the fmall-pox. His inquiries led him to fee how it was practifed in the Inoculation Hofpital, from whence he came away fo much prejudiced againft it, that he immediately inoculated one of his children with the fmall-pox. Soon after he found the opinions he had taken up, to arife from the vaccine having mixed itfelf with the variolous infection in this hofpital; and his further inquiries ended in a perfect conviction of the merits of vaccine inoculation, infomuch that he inoculated another of his children with it, who went through the difeafe perfectly well, and has fince refifted the variolous infection, which was attempted to be communicated feventeen months after the other. He attributes the difcovery folely to Dr. Jenner. In his official fituation he recommended to the Admiralty to have it tried on board the fleet, which was done, and in the Kent man of war all thofe who had received the vaccine inoculation, were afterwards inoculated with variolous matter, but without effect; that the Reports of all the Navy Surgeons were favourable to its operations; remarking, that the men, during the diforder, were not incapacitated from their ufual duties; and fo highly did they prize this difcovery, that a meeting was held at Plymouth, where a gold medal was fubfcribed for by them, and prefented to Dr. Jenner as its author. He laid before the Committee teftimonials from Egypt, figned by Lord Keith and General Hutchinfon, in its favour; his own opinion of its advantages, he draws from the comparifon with the mortality occafioned by the fmall-pox, which, by computation, amounts to nearly one-tenth of the whole mortality in this country (95 out of every 1000 deaths reported in the bills); and that fince the introduction of inoculation for the fmall-pox, the mortality occafioned by that difeafe has increafed; that the number who die of the fmall-pox annually within the bills of mortality is about 2000, and in the United Kingdoms it may be computed at 45000; therefore if the vaccine was univerfally fubftituted, he thinks the fmall-pox muft in a fhort time be extinct; he has heard of objections and prejudices againft this method, but upon inquiry he has found them grounded on fallacy and mifreprefentations; an inftance of which occurred in the 10th regiment of dragoons, where he found the lancets ufed to have been confounded with others armed with variolous matter, which probably occafioned the report of fmall-pox infection having fucceeded the inoculation with vaccine matter. He gave two or three other inftances equally injurious to the practice of vaccine inoculation, which were evidently founded upon mifapprehenfion. He believes moft of thefe cafes to have arifen from the ufing of matter taken at too late a period of the puftule, which may may equally happen in inoculating for the fmall-pox with virus taken at an improper period of maturation. (No. 4.)

Dr. BRADLEY, Licentiate of the Royal College of Phyficians, Phyfician to the Weftminfter Hofpital, and one of the Conductors of the Medical and Phyfical Journal, is in correfpondence with the Faculty on the Continent as well as in England; has received accounts of the progrefs of vaccine inoculation from New York and Philadelphia; from Paris, Malta, Italy, and Germany; from all which parts its excellence is confirmed, and it is now likewife introduced into Turkey. He laid before the Committee all the publications and papers he had received on that head. He looks upon Dr. Jenner as the author of the vaccine inoculation, and believes no medical man in the world doubts it; and in his extenfive correfpondence he has never heard any other perfon lay claim to it; he believes vaccine inoculation will prevent the fmall-pox to the extent of human life; for the natural cow-pox has already been proved to do fo; and there have been decifive experiments made to prove that vaccine inoculation will mitigate the fmall-pox, when caught in the natural way. The fpurious fort of cow-pox can be readily diftinguifhed from the real, by an examination of the plates given as illuftrations of the practice by Dr. Jenner; he thinks that if Dr. Jenner had fettled in London, and kept the practice a fecret, he might have made £.10,000 per annum for the firft five years, and double that fum afterwards; for notwithftanding the affiduous labour of Dr. Jenner and others to inftruct practitioners, important errors are daily committed in it, both at home and in foreign parts. He believes that not lefs than two millions of perfons have been inoculated with vaccine matter in the world, and he has never known one inftance of a patient dying in confequence of this mode of inoculation; and he has only *heard* of four cafes which were faid to have failed, to the explanation of which Dr. Woodville, Mr. Cline, and Mr. Ring can fpeak. He believes the computation of deaths occafioned by inoculated fmall-pox, to be one in three hundred, in England; and not lefs than one in one hundred and fifty, throughout the reft of Europe, Africa, Afia, and America. (No. 35.)

Dr. WOODVILLE, Licentiate of the Royal College of Phyficians, and Phyfician to the Small-pox Hofpital, confiders Dr. Jenner as the original difcoverer of vaccine inoculation. He has introduced it in one of the hofpitals under his care, in confequence of the communications of Dr. Jenner. He gives the preference to the vaccine over the fmall-pox inoculation, becaufe he finds it equally certain in fecuring the patient from the fmall-pox, becaufe it is without danger or rifk of life, and not, like the fmall-pox, contagious. One patient in the hofpital was faid to have died of the vaccine inoculation, but in his opinion it was not fo, as he had previoufly caught the fmall-pox in the natural way, to which his death ought to be attributed; the cafe of failure which Dr. Bradley mentioned, was a child who had been inoculated with the cow-pox, but who died in confequence of a bowel complaint, attended with diarrhœa to fo violent a degree that he attributed its death to that diforder, and not to any thing belonging to vaccine inoculation. He has inoculated 7500 patients up to laft January with the vaccine difeafe, about half of which number have been fince inoculated with fmall-pox matter, in none of whom did the fmall-pox produce any effect. The mortality occafioned by the fmall-pox will be found in the calculation delivered in; which agrees with Dr. Blane's. (No. 3.)

Sir WALTER FARQUHAR, Bart. Licentiate of the Royal College of Phyficians, and Phyfician to his Royal Highnefs the Prince of Wales, ftated, That he never heard of vaccine inoculation previous to its introduction by Dr. Jenner. Two of his own grand children were inoculated at the fame time; one with the fmall-pox in the ufual manner, who had it at firft in a favourable manner, but latterly attended with confiderable eruptions and convulfion fits; the other child was inoculated with the cow-pox, which he underwent in the mildeft manner poffible, and on the 12th day from the inoculation was brought home to his brother, and lived with him during the progrefs of the fmall-pox, without the fmalleft fymptoms of catching it. He confiders vaccine inoculation as the greateft difcovery which has been made for many years; thinks Dr. Jenner has fuffered in his fortune materially by making this difcovery public; that on its firft being communicated to him by Mr. Cline, he faid, that if Dr. Jenner was confident of its fuccefs, and would refide in London, he would infure him £.10,000 per annum; but that if he fuffered the fecret to be divulged, every practitioner would get hold of it, and Dr. Jenner lofe all chance of emolument. This has actually happened, and he has therefore loft the opportunity of making his fortune. He is of opinion that vaccine inoculation is a permanent fecurity againft variolous infection, and it never has proved fatal. The general computation of the mortality of the fmall pox, when performed in the beft manner, is about one in three hundred. (No. 36.)

Mr. JOHN RING, Member of the Royal College of Surgeons, confiders Dr. Jenner as the author of vaccine inoculation, and the difcovery itfelf as being beyond all comparifon the moft valuable and important ever made by man; he believes it to to be a perfect and permanent fecurity againft the fmall-pox; he has inoculated upwards of 1,200 perfons with vaccine matter, and has reafon to believe that at leaft 1,000 of them have been either voluntarily or involuntarily expofed to variolous infection, which they all refifted. The vaccine inoculation is attended with no danger unlefs from ignorance or neglect. If Dr. Jenner had kept this difcovery to himfelf, his practice might have been worth £. 10,000 per annum, it being well known that certain individuals have acquired as much or more by the ordinary practice of phyfic; all humours and diforders which happen after any fpecies of inoculation, are commonly attributed to that inoculation by perfons prejudiced againft it, and others are fometimes influenced by their opinions; but he knows of no inftance where the cow-pox has occafioned any other complaint, than what may be caufed by any other difeafe which is equally mild; he is of opinion that every difeafe is capable of exciting other difeafes or humours, in proportion to its magnitude; the magnitude of the cow-pox depends much upon the treatment. He never practifed the fmall-pox inoculation in any particular manner, nor ever kept any account of the number he inoculated, but fuppofes it might amount to about 600; he thinks that about one in every hundred in London, on an average, inoculated with fmall-pox, die; the reafon of a greater mortality prevailing amongft perfons inoculated for the fmall-pox in London, is the unwholefomenefs of the atmofphere, and the frequent neceffity of inoculating children at an improper age; he has never known any accident happen from inoculating from a fpurious fort of cow-pox; in refpect of the periods of coming out and turning, the inoculation of the cow-pox is fubject to the fame laws, and liable to the fame variations, with the inoculation of the fmall-pox; it is not more difficult to determine whether a patient has had the regular cow, than whether the patient has had the regular fmall-pox, provided care is taken not to interrupt the regular progrefs of the vaccine puftule by friction; he has known local inflammation produced both from inoculation with vaccine, and inoculation with variolous matter, without being followed by any puftule; in this refpect, therefore, the two inoculations are fimilar, and he knows of no advantage either in this or any other refpect which the inoculation of the fmall-pox has over that of the cow-pox. (No. 38.)

Dr. JAMES SIMS, Licentiate of the Royal College of Phyficians, and Prefident of the Medical Society of London, ftated, That he was originally adverfe to vaccine inoculation, but his confidence had been increafing in it every hour, from the repeated trials and authorities cited of its efficacy. He never heard heard of it before Dr. Jenner's publication, to whom alone he attributes the difcovery, which he looks upon to be the moft ufeful ever made in medicine; he thinks that if Dr. Jenner had kept it a fecret, as he might have done, he might, during his life (if protracted to a moderate length) have become the richeft man in thefe kingdoms. The vaccine difeafe does not introduce any other diforder into the human frame. The computation made of deaths occafioned by the natural fmall-pox, by Dr. Jurin and others, is one in fix.

Dr. Sims laid before the Committee a teftimony, unanimoufly refolved upon by the Medical Society of London, (which confifts of above 150 members refident in the metropolis, and of more than double that number refiding elfewhere) in favour of this very important difcovery, figned by himfelf as prefident. (No. 37.)

Mr. JOHN ADDINGTON, Member of the Royal College of Surgeons, is acquainted with vaccine inoculation, and has practifed it fince the fpring of the year 1799 with uniform fuccefs, and has kept an exact regifter of cafes to the number of eighty-one, with all their particulars. He has inoculated with variolous matter, and expofed to the infection of natural fmall-pox in its moft violent forms, and in every ftage, by every method he could devife, about one-third of his patients, and in no cafe was the infection of fmall-pox communicated. He further ftated, that he had been particularly careful in the choice of the matter employed in vaccine inoculation, and had not found in his own practice any cafe of fpurious cow-pox, but had feen many cafes of fpurious fmall-pox; and therefore confidered that the objections which are thought to arife againft the vaccine inoculation from this fource, apply equally againft the inoculation of fmall-pox. (No. 21.)

Dr. LETTSOM, F. R. S. Licentiate of the Royal College of Phyficians, and Phyfician Extraordinary to the City of London Lying-In Hofpital, ftated, That he looked upon Dr. Jenner to be the difcoverer of vaccine inoculation. He thought that inoculation of the fmall-pox had increafed the number of deaths. About the year 1773, he paid particular attention to this fubject, which afforded fome obfervations applicable to the prefent inquiry, and decifive upon a large fcale of calculation, which a table by figures more clearly evinced. The experience of forty-two years preceding the introduction of inoculation into this country, was already placed in a clear point of view in the Philofophical Tranfactions, by Dr. James Jurin, who was a fanguine advocate for inoculation, and whofe teftimony was therefore unexceptionable. His numbers were taken from from the yearly bills of mortality, and the reaſon why the fourteen years from 1686 to 1701 were omitted, was, becauſe in the bills of thoſe years the account of the ſmall-pox and meaſles were not diſtinguiſhed as in the preceding and following years, but were joined together in one article, ſo that from them no certain account could be drawn of the number of perſons that died of the ſmall pox. It appeared by theſe tables, that out of 1,005,279 burials within the laſt forty-two years, 1,742 perſons more have died of the ſmall-pox than the proportionate number, as collected from the experience of the firſt forty-two years; ſeventeen more burials therefore in one thouſand had been occaſioned by the ſmall-pox, ſince inoculation had been adopted. He believes that the inoculation of the cow-pox ſecures the perſon inoculated from the ſmall-pox, as much as the method of inoculation for the ſmall-pox, with this difference, that the cow-pox is not infectious. Vaccine inoculation has diminiſhed the fatality occaſioned by the natural ſmall-pox, by leſſening the number ſuſceptible of taking it. Taking London and the out pariſhes as containing nearly 1,000,000 of people, he calculates, that 3,000 probably died yearly by the ſmall-pox, or eight every day; or allowing Great Britain and Ireland to contain 12,000,000 of people, no leſs than 36,000 annually. About eight perſons die by the ſmall-pox every day in the metropolis and its environs, or about fifty-ſix in each week; although, from ſome defects in the Bills of Mortality, the amount does not appear to have exceeded forty-five; But if he calculated the laſt three weeks in March laſt, which amounted to thirty-five deaths, and compared them with three weeks in March for ten years preceding, which amounted to 697, it would reſult that the preſent month was thirty-five leſs than the average of ten preceding years, that is, from 1790 to 1800. He thought that the genuine cow-pox was never fatal; he had reaſon to conclude that about 60,000 perſons had been inoculated with cow-pox. He had heard curſorily of four deaths; but upon minute inquiry, he was convinced that three of them had no connection with the cow-pox: Of the fourth, he had received no accurate information. But ſuppoſing the cow-pox, during its progreſs, to occupy fourteen days, it appeared by the deaths in London, that on a common average, in every 60,000 healthy ſubjects, ſeven died in fourteen days, without the infliction of any diſeaſe, but what was in the common courſe of events; that knowing the fatality of ſmall-pox, and riſk occaſioned by inoculation, he was early inquiſitive upon this important ſubject, both from its conſequence to mankind in general, and from his acquaintance with ſome particular families, who had ſuffered both from the natural ſmall-pox and inoculation; tion; and from thofe who had adopted the practice, no one unfavourable event has refulted. Hence he acquired the moft favourable opinion of the practice, which his fubfequent experience has not altered: That he had not known any inconveniences to follow the fpurious fort of cow-pox. He further ftated, That if Dr. Jenner had kept this practice a fecret to himfelf, he might have derived immenfe pecuniary profits; and that confidering the apparent incredibility of the practice to common obfervation, and the fecrecy with which the Suttonians long monopolized the inoculation of fmall-pox, that Dr. Jenner might have exclufively kept the practice to himfelf for a long period. Upon being afked, Whether he had ever known a patient who had been inoculated for the fmall-pox, undergo that difeafe a fecond time? he replied, That he had two relations inoculated under the Suttonian method, both of whom afterwards took the fmall-pox in a natural way, one of whom died; and lefs than twelve months ago, he had attended two children in different families, the parents of which affured him that they had been inoculated for the fmall-pox a year or two before his attendance, when both were attacked feverely with the natural fmall-pox. He added, that the mode of fmall-pox inoculation practifed by the Suttons and Baron Dimfdale, was the fame as now adopted; but by fome vague pretenfions of the former, the public opinion ran very generally in their favour, till Baron Dimfdale publifhed his account of the Suttonian method. (No. 41.)

Mr. CLINE, Member of the Royal College of Surgeons, and Surgeon to St. Thomas's Hofpital, ftated, That in July, 1798, he received fome vaccine matter from Dr. Jenner, with which he inoculated a boy who had not had the fmall-pox; when he had gone through the ftages of vaccine inoculation, he tried to infect him with fmall-pox by inoculation, but in vain; this circumftance, together with the communications he received from Dr. Jenner, produced the ftrongeft conviction in his mind of the great utility of this practice, and he therefore recommended it ftrongly to all his friends, amongft whom was Sir Walter Farquhar, and he perfectly recollects the converfation relative to the emolument Dr. Jenner might derive from the practice of vaccine inoculation; but Dr. Jenner at that time declined fettling in London. Mr. Cline looks upon it as the greateft difcovery ever made in the practice of phyfic, for the prefervation of the human race, as the fmall-pox had been the moft deftructive of all difeafes.

[ To be continued. ]

